# The Sernbo score as a predictor of 1-year mortality after hip fracture: a registry study on 55,716 patients

**DOI:** 10.1007/s00068-020-01375-4

**Published:** 2020-05-03

**Authors:** Carl Mellner, Margareta Hedström, Ami Hommel, Olof Sköldenberg, Thomas Eisler, Sebastian Mukka

**Affiliations:** 1 Department of Surgical and Perioperative Sciences, Umeå University, Umeå, Sweden; 2 Department of Clinical Science, Intervention and Technology (CLINTEC), Karolinska Institutet, Stockholm, Sweden; 3 Department of Care Science, Faculty of Health and Society, Malmö University, Malmö, Sweden; 4 Department of Clinical Sciences at Danderyd Hospital (KIDS), Division of Orthopaedics, Karolinska Institutet, Stockholm, Sweden

**Keywords:** Hip fracture, Femoral neck fracture, Elderly, Mortality, Score

## Abstract

**Purpose:**

Patients sustaining a hip fracture have a high mortality rate during the first postoperative year and the Sernbo score may stratify patients into a high, intermediate and low risk of death during this period. We assessed its predictive properties on patients from the National Swedish Hip Fracture Register.

**Patients and methods:**

55,716 hip fracture patients, 69% women older than 65 years at surgery (registered between 2010 and 2015) with complete Sernbo scores and mortality data were studied. Receiver-operating characteristics analyses (ROC) were used. Validation of Sernbo score was performed.

**Results:**

The overall 1-year mortality rate was 26%—and 17%, 27.4% and 55.6% in the low, intermediate and high-risk groups, respectively. The ROC analysis indicated a predictive ability of the Sernbo score, with an AUC of 0.69 (CI 0.68–0.69).

**Conclusion:**

In this registry-based study, the easy-to-use Sernbo scoring system proved to be appropriate and useful way to identify hip fracture patients with a high-risk mortality during the first postoperative year.

**Electronic supplementary material:**

The online version of this article (10.1007/s00068-020-01375-4) contains supplementary material, which is available to authorized users.

## Introduction

An increasingly large and frail aging population are at risk for fragility fractures of the hip, which is associated with a high morbidity and 1-year mortality [1]. There are a number of scores predicting the postoperative mortality that rely on accurate definition of comorbidity and formulaic calculations. These scoring systems are invented to identify patients at risk for early mortality and may provide a possibility to optimize patients pre- and post-operatively. The most commonly used are POSSUM [2, 3], the Charlson Comorbidity Index [4] and Nottingham hip fracture score [5]. Previously, the Sernbo score has been found to be a useful predictor of 1-year mortality in a small cohort of patients with femoral neck fracture [6]. The Sernbo score is a simple four-component score (age, habitat, walking aids and mental state), initially developed as a tool for decision making for treatment with either a total- or hemiarthroplasty for femoral neck fractures [7, 8]. The aim of this study was to validate Sernbo score on a national level. The Sernbo score can easily be calculated using information obtained during routine orthopaedic patient assessment.

## Patients and methods

### Study design

This retrospective cohort study included all patients above the age of 65 years old, surgically treated due to a hip fracture between 2010-01-01 and 2015-12-31 registered in the Swedish Hip Fracture Register (SHFR) [9–11]. The guidelines of the STROBE (STRrengthening the Reporting of OBbservational studies in Epidemiology) statement were followed [12]. Validation of the Sernbo score using the SHFR.

## Source of data and terminology

In the Swedish Hip Fracture Register (SHFR), patients with hip fractures treated in Sweden have been registered since 1988. In 2016, the completeness of SHFR was 84% [13]. Baseline data on all patients include age, sex, ASA category (1–2 or 3–5), pre-fracture walking ability, habitat, cognitive status and type of fracture. We classified walking ability as either with or without any walking aid. Habitat, as either living independent or at a sheltered home. In the current study, cognitive status was classified as lucid or cognitive impairment and the type of fracture as femoral neck or trochanteric/subtrochanteric.

Date of death was obtained through record linkage with the National Death Register. In Sweden, The National Register of Causes of Death is cross-checked continuously with the National Death Register. All data were linked to the patients using the unique personal identification number assigned to all Swedish citizens. The Sernbo score was graded according to the total number of points and divided into low risk (17–20 points), intermediate risk (14 points) and high risk (8–11 points), as described previously Mellner et al. [6] (Table 1).

**Table 1:** The Sernbo score was graded according to the total number of points and divided into low risk (17–20 points), intermediate risk (14 points) and high risk (8–11 points)

Factor	Points	Mortality 1 year tested separately
Age		
< 80 years	5	4.6%
≥ 80 years	2	22.8%
Social situation		
Own home	5	13.6%
Sheltered home	2	13.8%
Walking aids		
None or one stick	5	6.8%
Two sticks or walking frame	2	20.5%
Mental status		
Alert	5	12.2%
Slight confusion	2	15.1%

### Statistical analysis

Descriptive data were presented with means and standard deviation (SD), range and percentages. A univariate and multivariate logistic regression analysis was performed to predict mortality. Associations were quantified using odds ratio (OR). ROC (Receiver-operating characteristic) curves were calculated to validate the mortality thresholds given by the Sernbo score and to determine their sensitivity and specificity. C-statistics (area under the curve, AUC) with 95% confidence interval (95% CI) was used to assess discrimination of the outcomes. The Hosmer–Lemeshow test were used to assess calibration of goodness-of-fit. Nagelkerke’s *R*^2^ were used for testing the predictive value. Significance level was set at 0.05 and all tests were two-tailed. Kaplan–Meier survival curves were used to compare the different groups with log-rank test. The statistical analysis was performed using SPSS Statistics software 24.0 for Mac (SPSS Inc., Chicago, IL) and using the MedCalc (Medcalc Software, Ostend, Belgium) for the ROC analysis.

### Ethics

The study was conducted in accordance with the ethical principles of the Helsinki Declaration and was approved by Regional Ethical Review Board at the Karolinska Institute (DNR: 2017/1088-31).

## Results

### Patients and descriptive data

**Fig. 1:**
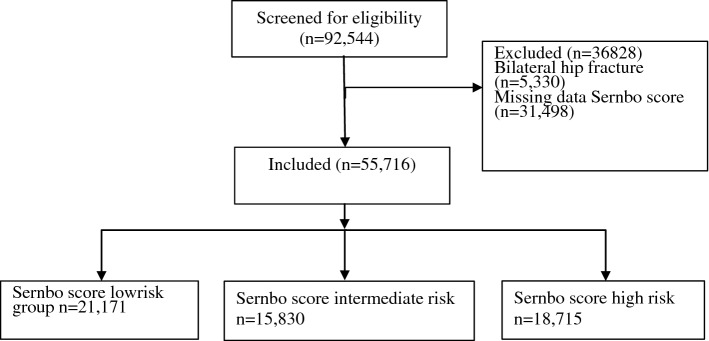
Flowchart of patients included in the study

During the study period, 87,214 patients (92,544 hips) were registered in the SHFR. 5330 patients (5.8%) sustained bilateral hip fractures during the study period, only the first fracture was included in the analyses. Patients with missing data regarding Sernbo score were excluded (*n* = 31,469) and 55,716 patients remained for analysis (Fig. 1). The mean age was 83 years (range 65–108) years and 69% were females (Table 2). 38.0% of patients were classified as low risk, 28.4% as having an intermediate risk while the remaining 33.6% formed the high-risk group.

**Table 2:** Patient demographics for all patients with hip fractures (*n* = 55,716)

Age	84 (65–108)
Sex	
Men	17,276 (31%)
Female	38,440 (69%)
1-year mortality	
Deceased	14,415 (26%)
Alive	41,301 (76%)
ASA	
1–2	23,505 (42%)
3–4	31,691 (57%)
Sernbo score	
High risk	18,716 (33.6%)
Intermediate risk	15,830 (28,4%)
Low risk	21,171 (38,0%)
Cognitive impairment	
Yes	5779 (64%)
No	19,937 (36%)

### Mortality

The 1-year mortality was 26% in the whole study group and 17%, 27.4% and 55.6% in the low, intermediate and high-risk groups respectively (log-rank test *p* < 0.001). A multivariable logistic regression analysis was performed including habitat (OR 2.0; 95% CI 2.0–2.2; *p* < 0.05), walking aids (OR 1.8 95% CI 1.7–1.9; *p* > 0.05), mental status (OR 1.8; 95% CI 1.7–1.9) and age (OR 1.7; 95% CI 1.7–1.9). Sernbo score (intermediate risk 1.7; CI 1.7–1.8; *p* < 0.001, high risk 3.6; CI 3.4–3.8; *p* < 0.001). The Hosmer–Lemeshow test for the multivariable logistic regression was significant (Chi^2^ = 167, *p* < 0.001, 8 degrees of freedom) and the Nagelkerke *R*^2^ was 0.151 (Table 3).

**Table 3:** Univariate logistic regression model 1-year mortality

**Sernbo score by group** (reference: low risk)			
Intermediate risk*	2.6	2.4–2.7	*p* < 0.01
High risk*	5.9	5.6–6.2	*p* < 0.01
**Sernbo score**			
**Social situation** (reference: own home)			
Sheltered home	3.4	3.3–3.6	*p* < 0.01
**Age** (reference: < 80)			
> 80 years	2.5	2.4–2.7	*p* < 0.01
**Walking aid** (reference: none or 1 stick)			
Two sticks or walking frame	2.6	2.5–2.7	*p* < 0.01
**Mental status** (reference: alert)			
Slight confusion	3.0	2.9–3.1	*p* < 0.01
**Age**	1.1	1.1–1.1	*p* < 0.01
**Sex** (reference: women)			
Men	1.7	1.6–1.7	*p* < 0.01
**Type of fracture** (reference: cervical)			
Trochanteric	1.02	0.99–1.06	*p* = 0.3
**ASA score** (reference: 1–2)			
3–4	3.0	2.8–3.1	*p* < 0.01

Low risk marked as reference value

### Receiver-operating characteristic curve analysis (ROC)

#### Sernbo score

**Fig. 2:**
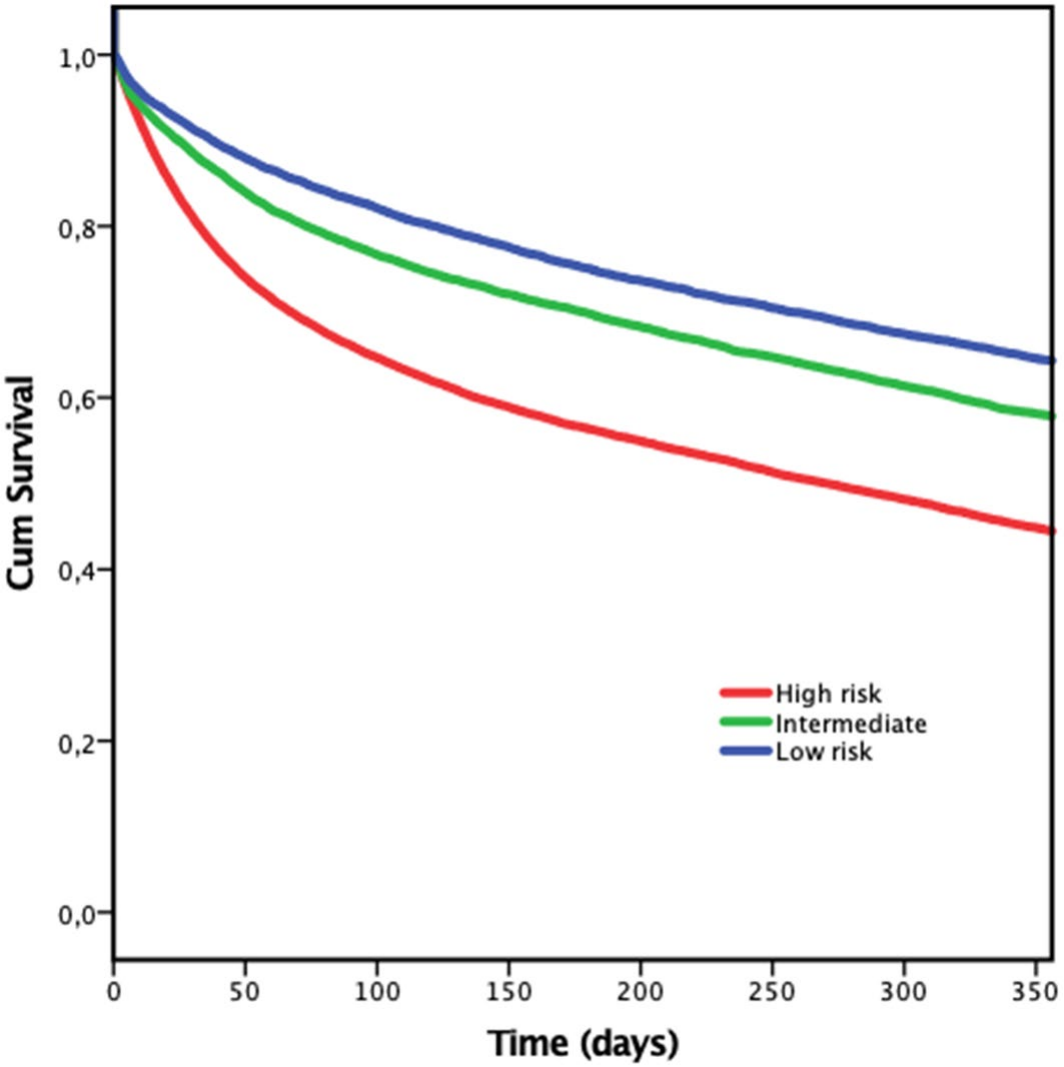
Kaplan–Meier survivorship curve displaying mortality

For 1-year mortality, the ROC curve analysis for the Sernbo score indicated a sensitivity of 83% and specificity of 54% (Fig. 2). Area under the curve (AUC) was 0.69 (95% CI 0.68–0.70) for 1-year mortality. Using each of the Sernbo components separately (i.e. age, habitat, walking ability and cognitive status) to predict 1-year mortality generated an AUC of 0.59, 0.64, 0.56 and 0.63, respectively. For the 30-day mortality, the AUC for modified Sernbo score was 0.68 (95% CI 0.68–0.70).

#### Generalizability

31,469 patients were excluded due to missing data. We found a slightly statistical age, but not clinically relevant difference in age, those included were slightly older 81.5 vs. 81.2 years (*p* < 0.01). We found a significant difference between those included and those excluded due to missing data, in 1-year mortality (26% vs 28%) (log rank test *p* < 0.01).

## Discussion

In this nationwide, retrospective cohort study based on the registry data, the Sernbo accurately identified patients with high risk of death during the first postoperative year. This easy, clinically applicable scoring system could be used more systematically to tailor pre- and post-operative care and might guide in the choice of surgical treatment (i.e. hemi- or total-hip arthroplasty) for patients with an acute hip fracture.

Our results in this large cohort of hip fracture patients corroborates the findings of Dawe et al., as well as a previous study from our department, both showing acceptable predictive abilities with an AUC of 0.69 and 0.79, respectively [6, 15]. The results suggested that the mortality in this group of patients is dependent on several additional unknown factors not included in these models. However, as the complexity of the scoring system increases, it might be at the expense of its clinical applicability. In this registry setting resulted in lower discriminative power compared to the more selected cohort at a single centre in Sweden [6]. Moreover, the latter study focused on a subpopulation of patients with displaced femoral neck fractures fit for hip arthroplasty surgery [6]. The Sernbo score was originally developed as a tool for decision making for treatment with either a THA or HA for displaced femoral neck fractures [7]. This might explain lower discriminative power when using Sernbo score on the whole hip fracture population. However, concurrent with our results, Söderqvist et al. [15] found no difference in mortality at 4 months and 24 months between the different types of hip fractures [15].

Karres et al., showed in a review of six scoring models for predicting 30-day mortality that none of the included models yielded neither good nor excellent discriminative power [16]. Trevisan et al. compared the survival rates between 2000 and 2015 and found that Charlson comorbidity index had the best predictive ability for mortality at 30 days and 1 year [17]. The Sernbo score seemed to be at par with most other predictive instruments in the literature [18].

The Charlson comorbidity index, Orthopaedic-POSSUM, Estimation of Physiological Ability and Surgical Stress and the Nottingham Hip Fracture Score, have all been evaluated as predictors of mortality in patients with hip fractures. In contrast to these scoring systems, the Sernbo score is far less complex which increases its daily clinical applicability [17]. In a recent publication, Jonsson et al., investigated the discrimination and accuracy of the POSSUM score, Portsmouth-POSSUM (P-POSSUM) score and the Nottingham Hip Fracture Score for prediction of mortality [19]. The authors found a moderate calibration and poor discriminative ability and concluded that mortality and morbidity in hip fracture patients are largely dependent on unknown factors that are not included in these scores [19]. The Sernbo score seems to perform on par with the above-mentioned scoring systems for mortality.

In the present study, we did not have data on co-morbidities, laboratory tests, such as haemoglobin or albumin on which other scoring system are based on i.e. O-POSSUM and NHFC [16]. In a publication by Karres et al. [16], presented an AUC of 0.78 in a fairly complex predicting model for 30-day mortality in hip fracture patients which included above 85 years of age, in-hospital fracture, signs of malnutrition, previous myocardial infarction, congestive heart failure, current pneumonia, chronic renal failure, malignancy and elevated serum urea.

Several other factors affecting the postoperative mortality in hip fracture patients have been reported, including the components in Sernbo score and fracture type, high ASA grade, high Charlson comorbidity score on admission, an abnormal ECG, increased C-reactive protein level, low haemoglobin level and hypoalbuminaemia [20–22]. Timing until surgery is associated with an increased mortality [23, 24]. A shorter length of hospital stay after hip fracture has been proposed to be associated with an increased 30-day mortality [25]. Previous studies have described a higher short-term mortality in males with a hip fracture [15, 27].

The major strength of the present study is the analysis of data from a large nationwide cohort of patients with hip fracture with a high degree of external validity by including patients with all types of hip fractures [28]. The unique personal identify number minimizes lost to follow-up and enables linkage to accurate mortality data at the National Death Register.

This registry-based, retrospective study design has inherited limitations. A limitation to the study is that it is not reproducible. We have not performed any test for reproducibility, such as interobserver and intraobserver reliability of each item. The exclusion of 30% of the registered patients due to missing data, mainly concerning cognitive function, which is an optional question in the registry. The assessment of calibration indicated a lower goodness of fit in our statistical models, we had a large sample size which might overestimate this issue. Poor calibration does not necessarily suggest a clinically relevant differences between prediction by the model and the observed outcome, we observed a fairly small, but still significant difference measured with the Hosmer–Lemeshow test [29, 30].

## Conclusion

In this registry-based study, the easy-to-use Sernbo scoring system proved to be appropriate and useful way to identify hip fracture patients with a high-risk mortality during the first postoperative year.

## Electronic supplementary material

Below is the link to the electronic supplementary material.

Supplementary file1 (DOCX 12 kb)

## Data Availability

The datasets used during the current study are not publicly available because of patient integrity, but are available from the corresponding author on reasonable request.
