# Supplementary material for: The Sernbo score as a predictor of 1-year mortality after hip fracture: a registry study on 55,716 patients
Source: Eur J Trauma Emerg Surg. 2020 May 3;47(6):2043–8. doi: 10.1007/s00068-020-01375-4 (PMC8629894; doi:10.1007/s00068-020-01375-4)
Supplement: Supplementary file 1 — Supplementary file1 (DOCX 12 kb) [file 68_2020_1375_MOESM1_ESM.docx]

**Supplemental Table 5.** Classification table for multivariate logistic regression illustrating observed and predicted mortality for 1-year mortality.

| **Classification Table** | | | | | |
| --- | --- | --- | --- | --- | --- |
|  | Observed | | Predicted | | |
|  |  |  | 1 year mortality | | Percentage Correct |
|  |  |  | No | Yes |  |
|  | 1 year mortality | No | 38,471 | 2,333 | 94 |
|  |  | Yes | 11,050 | 3,195 | 22 |
|  | Overall Percentage | |  |  | 76 |
